# Functionalized Biomimetic Scaffolds for Human‐Derived Auditory Neural Circuit Construction

**DOI:** 10.1002/advs.76525

**Published:** 2026-07-11

**Authors:** Pan Feng, Qian Zhu, Yusong Wang, Hao Rong, Xu Zhang, Fuchun Wang, Tianqi Yu, Wenxuan Wang, Jing Li, Lei Tian, Menghui Liao, Renjie Chai, Yangnan Hu

**Affiliations:** ^1^ Spine Surgery Department Nantong First People's Hospital State Key Laboratory of Digital Medical Engineering Jiangsu Provincial Key Laboratory of Critical Care Medicine School of Life Sciences and Technology School of Medicine Advanced Institute for Life and Health Southeast University Nanjing China; ^2^ Department of Rehabilitation Medicine Zhongda Hospital Southeast University Nanjing China; ^3^ The affiliated Lihuili Hospital of Ningbo University Ningbo China; ^4^ School of Medical Engineering, Affiliated Zhuhai People's Hospital Beijing Institute of Technology Zhuhai China; ^5^ Co‐Innovation Center of Neuroregeneration Nantong University Nantong China; ^6^ Department of Neurology, Aerospace Center Hospital, School of Life Science Beijing Institute of Technology Beijing China

**Keywords:** auditory neural circuit, biomimetic scaffolds, hydrogel, human induced pluripotent stem cell, spiral ganglion neuron

## Abstract

Damage to auditory circuits results in sensorineural hearing loss. However, the scarcity of human inner ear tissue significantly hinders the development of therapies to preserve auditory function, creating a critical need for reliable in vitro models. While human‐derived neural circuits offer therapeutic promise, generating high‐purity, functionally mature spiral ganglion neurons (SGNs) and achieving their oriented integration remain substantial challenges. In this study, we successfully differentiated human induced pluripotent stem cells (hiPSCs) into SGN‐like neurons that closely resemble in vivo counterparts. Using electrically conductive biomimetic scaffolds with highly ordered topological structures promoted SGN‐like neurons maturation, growth orientation. Furthermore, by co‐culturing SGN‐like neurons with denervated cochlear tissues, we established a human‐derived in vitro model capable of mimicking the auditory neural circuit. We efficiently recapitulated an in vitro auditory neural circuit on biomimetic scaffolds, elucidated transcriptional changes underlying SGN‐like neurons' maturation, and reproduced the protective phenotypes against cisplatin‐induced auditory circuit damage. These results demonstrate that the constructed human‐derived in vitro neural circuit model is a reliable platform for drug screening with broad application prospects.

## Graphical abstract

In Vitro Auditory Circuit Model.

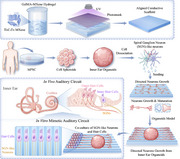

## 1 Introduction

Hearing loss is one of the most prevalent sensory impairments worldwide [1]. The World Health Organization estimates that by 2050, nearly 2.5 billion people will have some degree of hearing loss [2]. The vast majority of cases involve sensorineural hearing loss, with causative factors including ototoxic medications [3, 4], noise exposure [5, 6, 7], and genetic mutations [8, 9, 10]. Damage to the auditory circuit, encompassing sensory hair cells, spiral ganglion neurons (SGNs), and their central synaptic connections, is a major cause of irreversible sensorineural hearing loss [11]. Disruption of this finely organized neural pathway not only impairs sound transduction and signal transmission but also leads to long‐term deficits in auditory perception and speech recognition. Despite significant advances in inner ear biology, the complex cellular composition, precise spatial organization, and activity‐dependent maturation of the auditory circuit remain difficult to recapitulate in vitro. Although animal models and 2D cell cultures have provided important insights into auditory system development and pathology, they fail to fully capture the human‐specific features of the auditory neural circuit [12, 13]. In particular, the absence of in vitro platforms capable of reconstructing functional, multi‐level auditory connectivity severely limits mechanistic studies of ototoxic injury and hampers the translation of candidate therapeutics into clinical applications.

**SCHEME 1:**
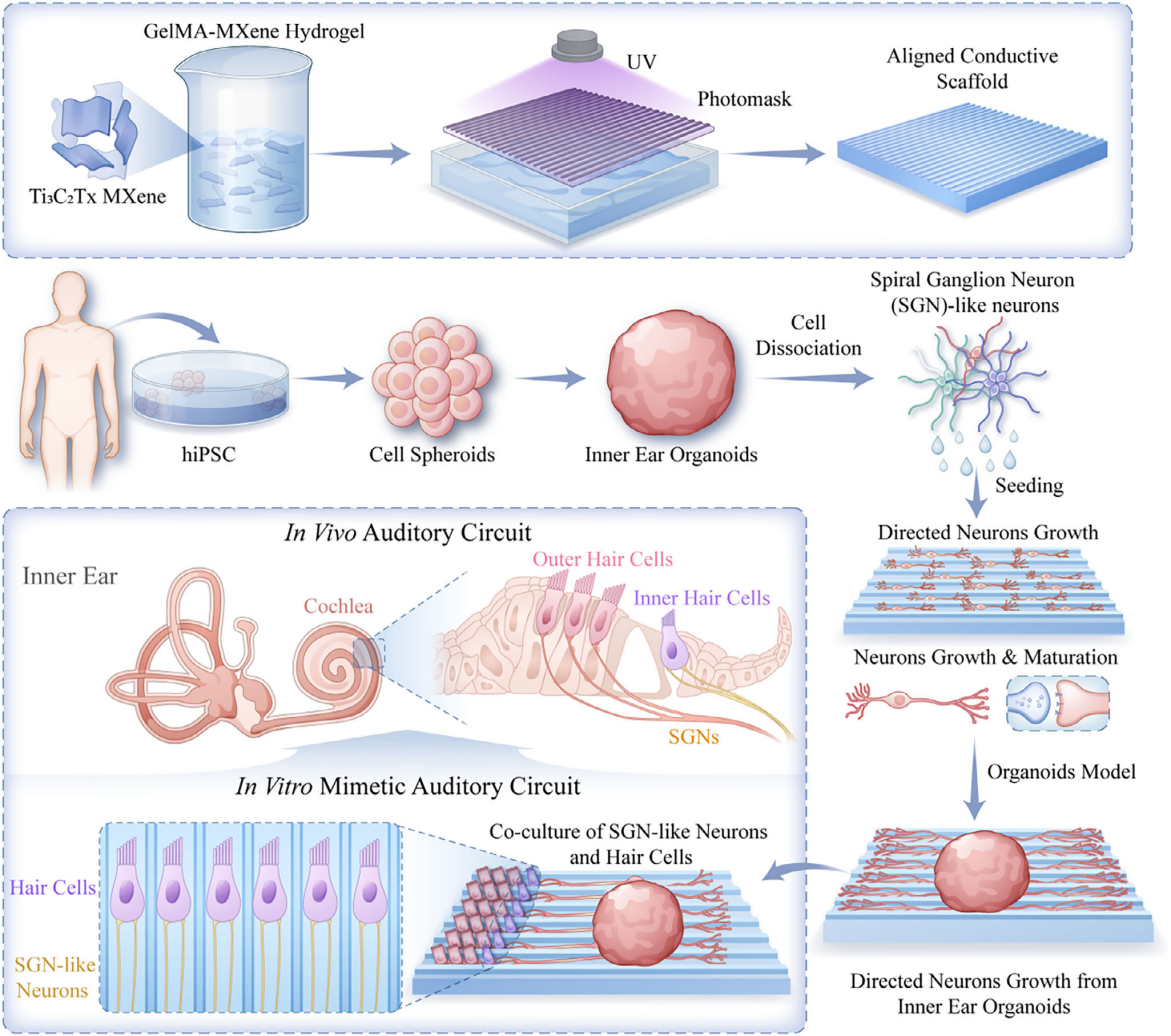
Schematic illustration of a humanized auditory circuit reconstructed using the biomimetic scaffolds.

Therefore, we propose a human‐derived auditory circuit model with defined topological organization and functional maturity, which can be applied to investigate protective mechanisms of the auditory circuit (Scheme 1). As the primary afferent neurons in the auditory pathway, SGNs relay signals from hair cells to the cochlear nucleus, establishing the essential link between the periphery and the central auditory system [14, 15, 16, 17]. Degeneration of SGNs, caused by excitotoxicity, neuroinflammation, or deficiency of neurotrophic factors, disrupts both the structural and functional integrity of this circuitry, ultimately leading to irreversible deficits in sound processing [18, 19, 20]. Such circuit‐level dysfunction presents a challenge for current therapeutic strategies, particularly in cases involving extensive SGNs loss. As mammalian SGNs have a very limited ability to regenerate naturally [21, 22], efficiently generating human‐derived SGNs is essential for reconstructing auditory circuits in vitro. In this context, directing hiPSCs towards SGN‐like neurons is a promising strategy for replacing damaged SGNs and promoting auditory nerve regeneration. Under defined in vitro culture conditions, hiPSCs can be efficiently directed toward diverse functional cell types relevant to inner ear repair. However, the direct induction of hiPSCs into SGN‐like neurons lineages remains challenging, particularly in achieving high efficiency and functional maturation. Since SGNs are organized in ordered patterns in the cochlea and project long distances to the cochlear nucleus throughout the auditory pathway [23, 24], successful regenerative repair is further constrained by the stochastic growth of newly generated neurons. Ensuring their precise integration into the auditory circuit therefore remains a fundamental challenge. Growing evidence indicate that bioengineered scaffolds offer significant advantages for functional integration, they are widely used to promote neural stem cell proliferation [25, 26], differentiation [27, 28] and overall functional integration [29, 30, 31]. These advances have laid a solid foundation for the construction of in vitro auditory neural circuit models that exhibit both structural and functional characteristics. Biomimetic scaffolds that integrate key auditory relay nuclei could provide a promising platform for studying hierarchical and directional signal transmission. However, further investigation and optimization are required before such scaffold‐based systems can be used to reconstruct complex auditory circuits in vitro.

Here, we propose and construct an in vitro auditory neural circuit model based on electrically conductive biomimetic scaffolds. By optimizing the inner ear organoid differentiation protocol [32], hiPSCs were efficiently differentiated into inner ear organoids containing abundant SGN‐like neurons. Furthermore, high‐purity SGN‐like neurons were obtained through subsequent purification and culture. The electrically conductive biomimetic scaffolds were developed by integrating GelMA hydrogel with Ti_3_C_2_T_x_ MXene nanomaterials, resulting in excellent biocompatibility, electrical conductivity, and a highly aligned topological structure. The scaffolds established an optimal microenvironment for SGN‐like neurons, facilitating directed axonal outgrowth while promoting both morphological development and functional maturation. By co‐culturing these SGN‐like neurons with the cochlear basilar membrane on biomimetic scaffolds, we efficiently generated a functionally mature, biomimetic in vitro model of the auditory circuit. Within the co‐culture system, SGN‐like neurons achieved directed projection toward hair cells under scaffold guidance and successfully formed functional synaptic connections, thereby preliminarily simulating auditory signal transmission. Furthermore, we applied this model to drug screening, successfully validating its feasibility and efficacy in discovering auditory protective drugs. This in vitro auditory neural circuit platform not only provides novel technical approaches for investigating SGNs connectivity, circuit dynamics, and injury repair mechanisms but also offers a reliable tool for drug screening and efficacy validation. More importantly, the electrically conductive scaffold‐based co‐culture system established in this study provides a human‐derived in vitro neural circuit model, offering a promising platform for the development and clinical translation of novel strategies for hearing loss prevention and treatment.

## 2 Results and Discussion

### 2.1 Generation of Human SGN‐Like Neurons From hiPSCs

**FIGURE 1:**
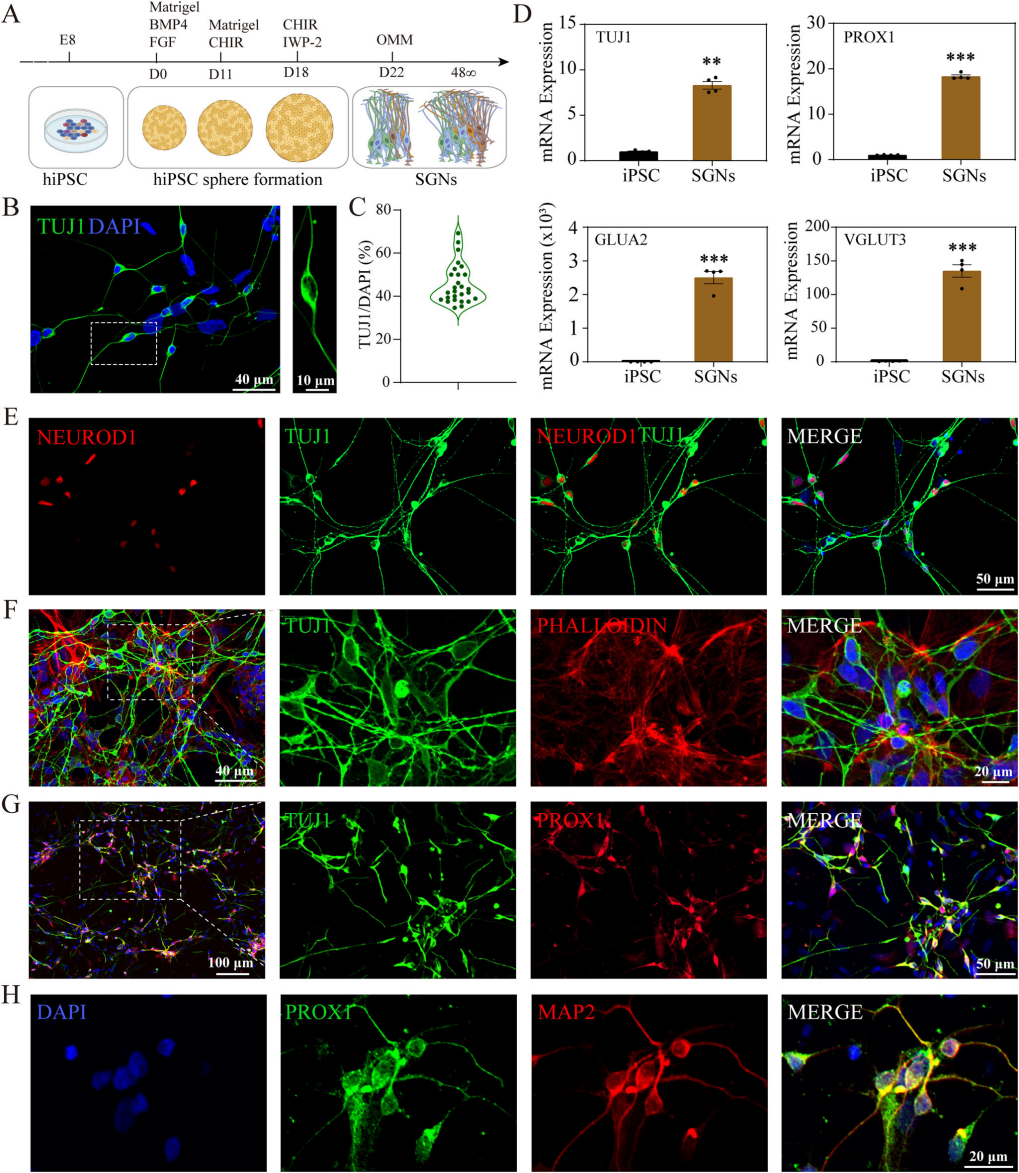
Differentiation and characterization of SGN‐like neurons from hiPSCs. (A) Overview and timeline of the induced differentiation from hiPSCs into SGN‐like neurons differentiation protocols; (B) Representative immunofluorescence images show TUJ1^+^ cells (green), DAPI (blue); (C) The proportion of TUJ1^+^ cells in the total cell population, used to indicate the differentiation efficiency of SGN‐like neurons in the total system; (D) qPCR validation of gene expression levels in SGN‐like neurons and hiPSCs; (E) Representative immunofluorescence images show TUJ1^+^ cells (green), NEUROD1^+^ (red), DAPI^+^ (blue); (F) Representative immunofluorescence images show TUJ1^+^ cells (green), PHALLOIDIN^+^ (red), DAPI (blue); (G) Representative images of immunofluorescence staining: neuronal marker TUJ1^+^ (green), SGN‐like neurons marker PROX1^+^ (red), and DAPI^+^ (blue). (H) Representative images of immunofluorescence staining: SGN‐like neurons marker PROX1^+^ (green), the mature neuron marker MAP2^+^ (red), and DAPI^+^ (blue). Data points illustrate the mean ± standard deviation (SD). *P*‐values are categorized as follows: ^**^ *p* < 0.01, ^***^ *p* < 0.001, and nonsignificant differences (*p* > 0.05) are designated as ns.

Here, to generate SGN‐like neurons, we recapitulated intercellular signaling during human inner ear development. By simulating cell‐cell communication [32, 33] and modulating key pathways, including BMP, FGF, WNT, and SHH in hiPSCs [34, 35], we established an approach for deriving inner ear organoids from single stem cell aggregates. This differentiation strategy, adapted from previous studies [32], efficiently directed hiPSCs toward the cochlear lineage under defined conditions. First, the formation of embryoid bodies was promoted under low‐adhesion culture conditions. By modulating the BMP, FGF, TGF, WNT, and SHH signaling pathways in a stepwise spatiotemporal manner, the embryoid bodies progressively differentiated into non‐neural ectoderm. This was followed by the formation of otic‐epibranchial progenitor‐like domains, which then developed into otic placode‐like structures. This process recapitulated the key stages of early inner ear development (Figure 1A). We dissociated the day 22 inner ear organoids into a single‐cell suspension using TrypLE, followed by plating them onto coverslips precoated with Matrigel. After several days of culture in neuronal maturation medium, cells exhibited typical neuronal morphology under bright‐field imaging (Figure S1). In this system, hiPSCs efficiently differentiate into SGN‐like neurons as evidenced by the expression of neuronal markers including NEUROD1, TUJ1, PROX1 [36] and MAP2 (Figure 1B,E–H and Figure S2A). SGN‐like neurons identity was further confirmed by the detection of the human neurofilament protein (hNFL), which revealed characteristic neurofilament‐positive neuronal projections (Figure S2B,C). These cells displayed a typical bipolar morphology with clearly extended axons and dendrites. Quantitative analysis showed that SGN‐like neurons accounted for over 50% of the total cell population (Figure 1C). Further comparison between SGN‐like neurons and hiPSCs by qPCR analysis showed that neuronal markers (TUJ1, PROX1, BRN3A, NEUROD1, PRPH [36] and ISL [37]) were significantly upregulated in SGN‐like neurons. Importantly, the expression of VGLUT3 [38], a vesicular glutamate transporter characteristic of glutamatergic SGNs, together with the glutamate receptor subunit GLUA2 [39], was also significantly increased, further supporting the acquisition of a glutamatergic SGN‐like phenotype (Figure 1D and Figure S3). The primer sequences used for qPCR are listed in Table 1. These results indicate that this differentiation system efficiently generates SGN‐like neurons with typical bipolar morphology and molecular profiles closely resembling those of native SGNs [36, 40, 41]. In summary, we successfully generated SGN‐like neurons by differentiating and dissociating inner ear organoids, as confirmed by multiple SGN‐associated markers. This system is a useful platform for studying SGN development, axon guidance, and auditory circuit reconstruction. However, this study primarily examined early‐stage SGN‐like neurons and did not systematically evaluate late maturation features, such as Schwann cell markers and myelination [42].

### 2.2 Integration of Biomimetic Scaffolds Enabling Directed Projection of SGN‐Like Neurons

**FIGURE 2:**
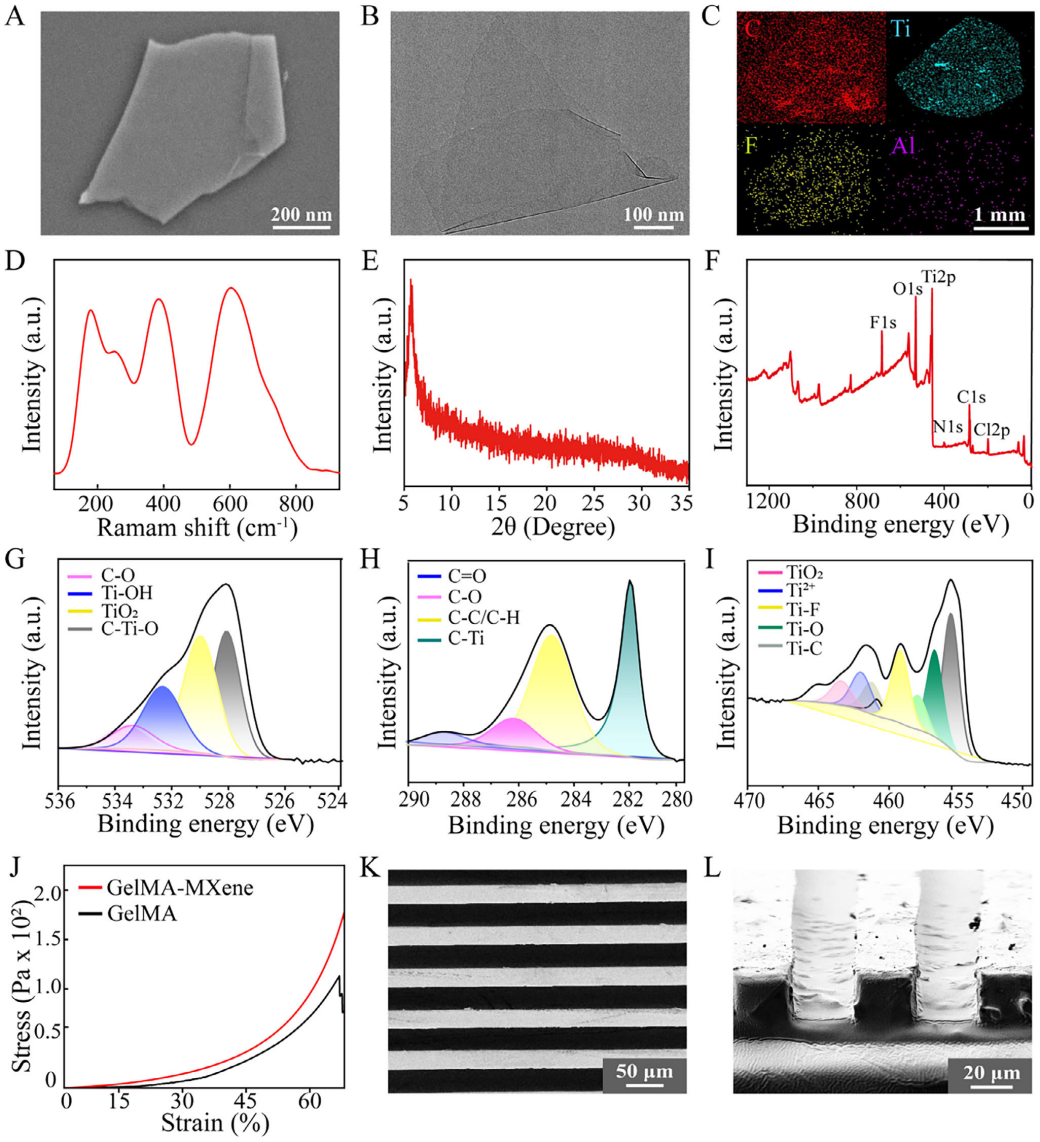
Characterization of MXene and GelMA‐MXene scaffolds. (A) SEM of MXene; (B) TEM of MXene; (C) EDX energy spectrum of MXene; (D) Raman spectra of MXene; (E) XRD diffraction peak of MXene; (F) XPS pattern images of MXene; (G–I) Fractional peak fit plots of the three elements C, O, and Ti in MXene; (J) MXene and GelMA‐MXene biomimetic scaffolds mechanical properties; (K) SEM of the GelMA‐MXene biomimetic scaffolds; (L) SEM side view image of the GelMA‐MXene scaffolds.

To induce directional growth of auditory neurons and reconstruct a human auditory circuit model, we developed an electrically conductive biomimetic scaffold with a defined topological architecture. The scaffolds, featuring a highly aligned topology, were fabricated by integrating GelMA hydrogel with Ti_3_C_2_T_x_ MXene, thereby combining the favorable biocompatibility of GelMA [43, 44] with the superior electrical conductivity of MXene [45]. To obtain monolayer MXene nanosheets, the aluminum layer of Ti_3_C_2_T_x_ was selectively removed using a LiF/HCl etchant. Electron microscopy revealed that the MXene nanosheets exhibited an average lateral size of 1–5 µm (Figure 2A,B). Subsequently, x‐ray diffraction (XRD), Raman spectroscopy, and x‐ray photoelectron spectroscopy (XPS) demonstrated the successful preparation of MXene. Energy‐dispersive x‐ray spectroscopy (EDX) further indicated that MXene is primarily composed of Ti and C (Figure 2C). Raman spectra exhibited characteristic peaks of Ti_3_C_2_T_x_ MXene in the range of 200–800 cm^−^^1^, with bands at 230–470 cm^−^^1^ attributed to surface functional groups associated with titanium atoms and a band at 580–730 cm^−^^1^ assigned to carbon‐related vibrations (Figure 2D). A distinct diffraction peak at 2θ = 6.4° corresponds to the characteristic lattice structure of MXene (Figure 2E). XPS analysis (Figure 2F–I) confirmed the characteristic elemental composition of MXene, primarily consisting of Ti, C, and O. Then, MXene was incorporated into the GelMA matrix to fabricate composite hydrogels. Subsequent stress‐strain analysis revealed that GelMA‐MXene biomimetic scaffolds exhibited superior mechanical properties compared to pure GelMA (Figure 2J). As visualized via SEM, the biomimetic scaffolds exhibited a highly aligned architecture across a large area (Figure 2K), further magnification confirmed that this orientation remained consistent, with uniform spacing maintained between adjacent structures (Figure 2L). Collectively, an electrically conductive GelMA‐MXene scaffold with a highly aligned topology was successfully fabricated. The scaffolds integrate biocompatibility, mechanical properties, and electrical conductivity. Importantly, the scaffolds present a well‐defined aligned architecture, providing a suitable platform for guiding directional neuronal growth and supporting the reconstruction of biomimetic auditory circuits.

### 2.3 Biomimetic Scaffolds Guided Outgrowth and Maturation of SGN‐Like Neurons

**FIGURE 3:**
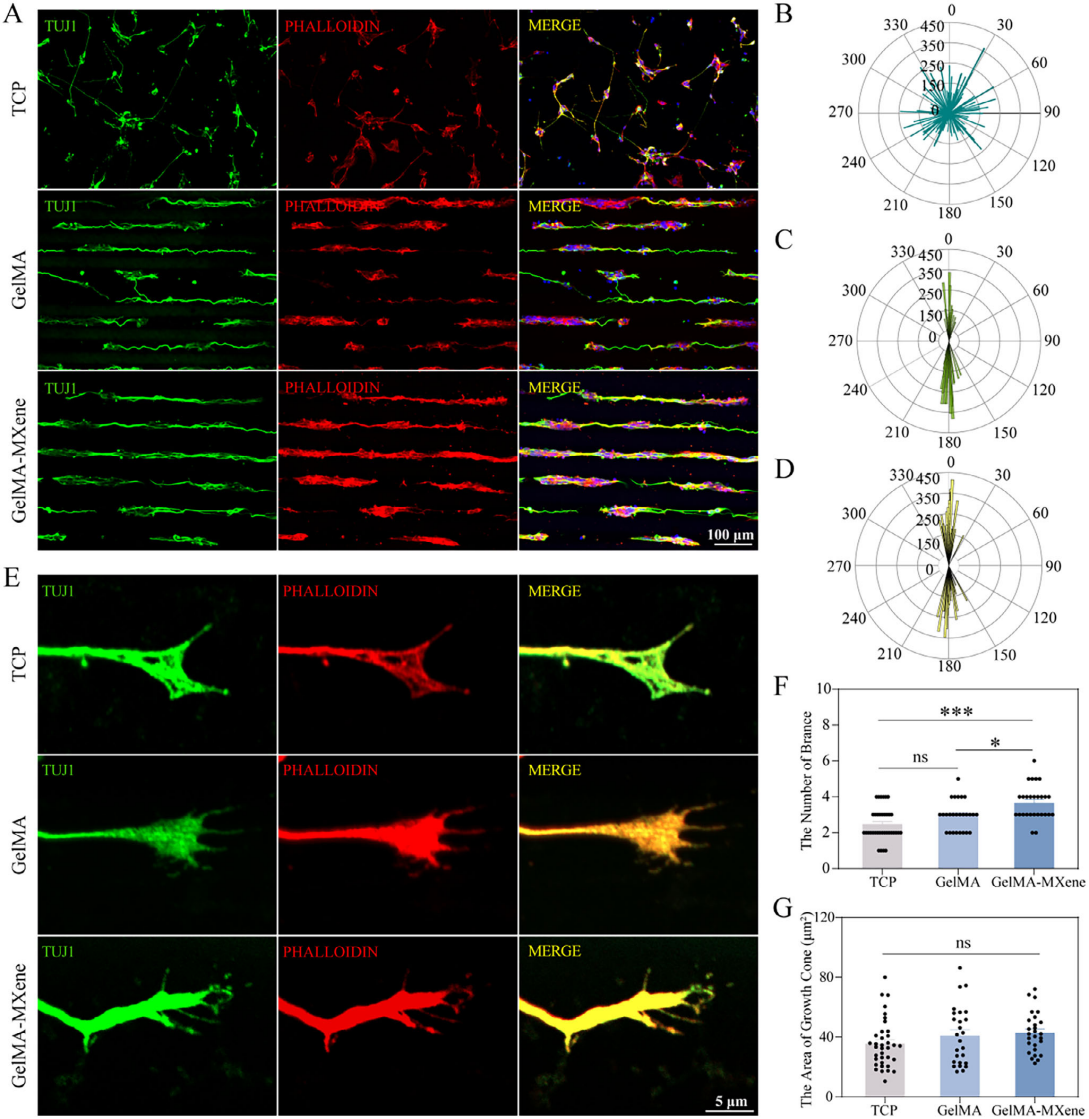
Axon‐directed growth of SGN‐like neurons with biomimetic scaffolds: (A) Representative confocal images showing TUJ1 and Phalloidin staining of SGN‐like neurons cultured on TCP and biomimetic scaffolds, TUJ1^+^ (green), PHALLOIDIN^+^ (red), DAPI^+^ (blue); (B) Distributional statistics of neural synapse orientation of SGN‐like neurons cultured on TCP; (C) Distributional statistics of neural synapse orientation of SGN‐like neurons cultured on GelMA biomimetic scaffolds; (D) Distributional statistics of neural synapse orientation of SGN‐like neurons cultured on GelMA‐MXene biomimetic scaffolds; (E) Representative confocal images showing the staining of SGN‐like neurons growth cones cultured on TCP, GelMA, GelMA‐MXene. TUJ1^+^ (green), PHALLOIDIN^+^ (red); (F) Statistics on the number of pseudopodia in SGN‐like neurons growth cones cultured on TCP, GelMA biomimetic scaffolds, and GelMA‐MXene biomimetic scaffolds; (G) Statistics on the area of pseudopodia in SGN‐like neurons growth cones cultured on TCP, GelMA biomimetic scaffolds, and GelMA‐MXene biomimetic scaffolds. Data points illustrate the mean ± standard deviation (SD). P‐values are categorized as follows: ^*^ *p* < 0.05, ^***^ *p* < 0.001 and nonsignificant differences (*p* > 0.05) are designated as ns.

Cochlear hair cells transmit sound signals to the cerebral cortex by forming ribbon synapses with SGNs [12, 46]. The directional projection of SGNs axons is critical for signaling between the cochlear sensory organs and the central auditory cortex. Therefore, we subsequently examined the effect of a biomimetic scaffolds with a highly aligned topology on the directional growth pattern of SGN‐like neurons. The biocompatibility of GelMA and GelMA‐MXene hydrogels was evaluated via CCK‐8 assays. While cell viability remained high at lower concentrations, a significant decline was observed as MXene levels reached 400 µg/mL. Consequently, a dosage of 300 µg/mL was selected for subsequent studies to ensure optimal cytocompatibility (Figure S4). To examine SGN‐like neurons growth patterns, the cells were cultured on Matrigel‐coated TCP, GelMA, and GelMA‐MXene biomimetic scaffolds. Matrigel was selected because of its favorable cell adhesion properties and extracellular matrix‐like composition, which provide a supportive microenvironment for the attachment, survival, and neurite outgrowth of SGN‐like neurons. After 5 days, TUJ1 and phalloidin staining revealed axonal orientation (Figure 3A). The results showed that SGN‐like neurons on TCP exhibited disorganized growth, whereas those on biomimetic scaffolds displayed a well‐aligned orientation. Quantitative analysis of axonal orientation (Figure 3B–D) showed that SGN‐like neurons on TCP exhibited a broad angular distribution (30°–330°), while those on biomimetic scaffolds were predominantly aligned around 0° and 180°. Over time, SGN‐like neurons on TCP became increasingly disordered, whereas cells on the biomimetic scaffolds maintained a more uniform and aligned orientation. Furthermore, SGN‐like neurons cultured on GelMA‐MXene biomimetic scaffolds exhibited significantly greater neurite extension compared with TCP and GelMA groups (Figure S5). Given that growth cones play a central role in axon guidance and extension [45, 47], we further analyzed growth cone morphology using TUJ1 and phalloidin staining (Figure 3E). MXene incorporation significantly increased the number of filopodia in SGN‐like neurons growth cones (Figure 3F), while no significant change in filopodial area was observed (Figure 3G). In summary, we developed a scaffold system that supports the directed growth of SGN‐like neurons. This system combines the biocompatibility of GelMA with the electrical conductivity of Ti_3_C_2_T_x_ MXene. The scaffold's ordered topological structure guides neurite extension. Additionally, the Matrigel coating provides an extracellular matrix‐like microenvironment that promotes the attachment, survival, and neurite growth of SGN‐like neurons. However, the system cannot yet fully replicate the complex cochlear microenvironment in vivo, and the long‐term biocompatibility of MXene requires further evaluation.

### 2.4 Biomimetic Scaffolds Promoted Synaptic Development and Calcium Activity of SGN‐Like Neurons

**FIGURE 4:**
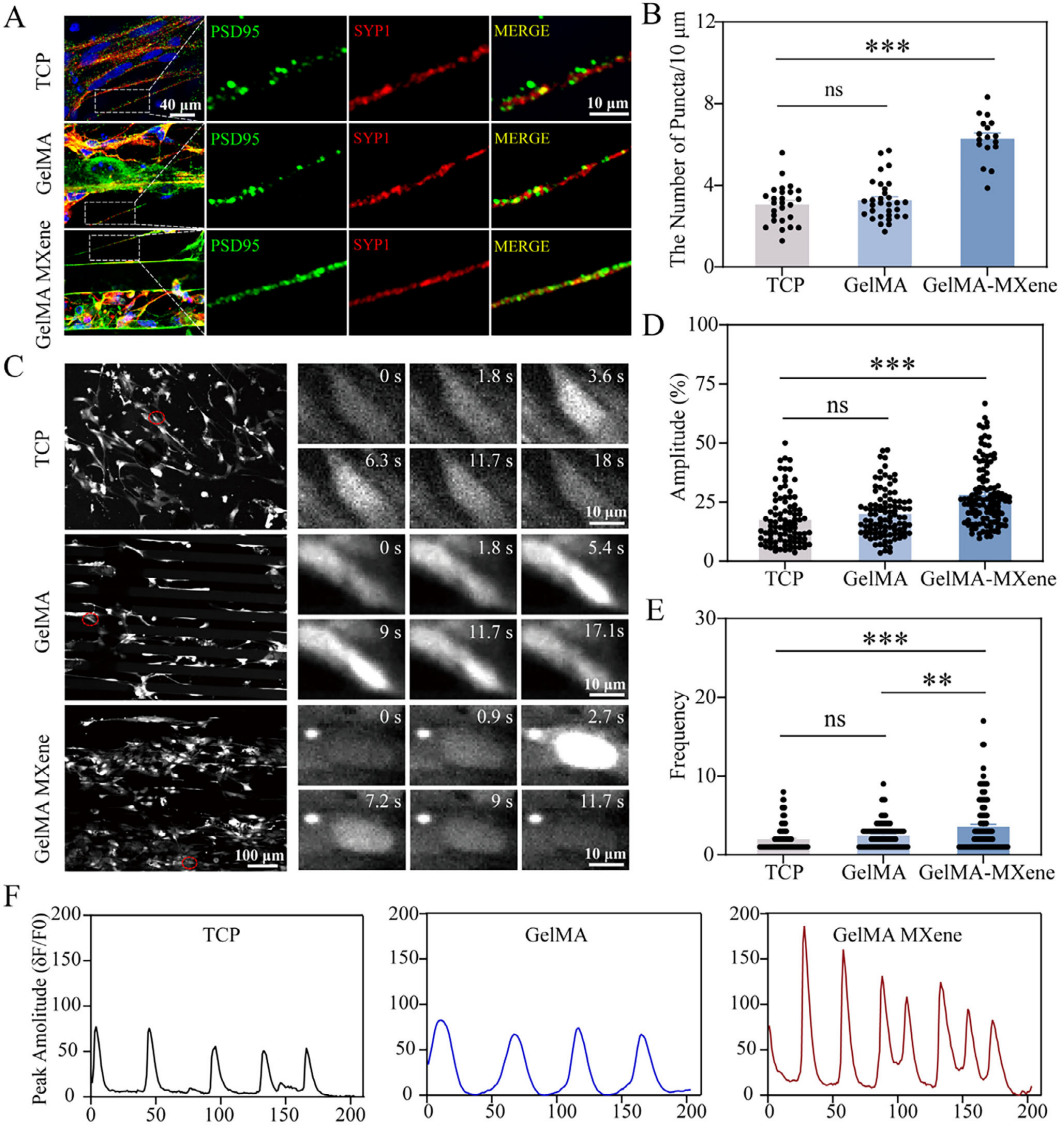
Functional maturation of human‐derived SGN‐like neurons in biomimetic scaffolds: (A) Representative confocal microscopy images of immunofluorescence staining of PSD95 and SYP1 in SGN‐like neurons cultured on TCP and biomimetic scaffolds, PSD95^+^ (green), SYP1^+^ (red), DAPI^+^ (blue); (B) Quantitative analysis of synaptic puncta density (per 10 µm) based on the colocalization of SYP1 and PSD95; (C) Schematic illustration of transient intracellular Ca^2^^+^ dynamics in SGN‐like neurons during calcium oscillations. Color intensity represents relative Ca^2^^+^ levels. A complete oscillatory sequence, segmented into six chronological intervals, is displayed on the right for an individual spiral ganglion neuron; (D) Quantitative analysis of calcium oscillation amplitudes. (E) Quantitative analysis of calcium oscillation frequency; (F) Representative calcium oscillation traces of SGN‐like neurons cultured on TCP, GelMA, and GelMA‐MXene biomimetic scaffolds. Data points illustrate the mean ± standard deviation (SD). *P*‐values are categorized as follows: ^**^ *p* < 0.01, ^***^ *p* < 0.001, and nonsignificant differences (*p* > 0.05) are designated as ns.

The conductivity of the biomimetic scaffolds was enhanced by incorporating MXene material into GelMA hydrogel. To evaluate whether the composite scaffolds maintain SGN‐like neurons electrical activity and promote functional maturation, synaptic density was assessed in neurons cultured for 14 days using immunofluorescence staining of postsynaptic density protein 95 (PSD95) and Synaptophysin 1 (SYP1). High‐magnification imaging revealed punctate and co‐localized expression of SYP1 and PSD95 along axons (Figure 4A). Analysis of marker expression and spatial distribution showed that GelMA‐MXene biomimetic scaffolds enhanced synaptic puncta formation (Figure 4B) and significantly upregulated PSD95 and SYP1 expression compared with controls (Figure S6A,B). These findings suggest that the biomimetic scaffolds promote synaptic maturation. Calcium imaging was performed to further assess SGN‐like neurons functional activity. Compared with TCP and GelMA groups, SGN‐like neurons cultured on GelMA‐MXene biomimetic scaffolds exhibited more dynamic Ca^2^^+^ oscillations. Specifically, the duration of individual oscillatory cycles was significantly reduced (Figure 4C), while both the amplitude and frequency were significantly increased (Figure 4D–F). Taken together, these results indicate that the GelMA‐MXene biomimetic scaffolds promote the maturation and functional refinement of SGN‐like neurons synapses via enhanced electrical conductivity.

### 2.5 Transcriptomic Insights Into MXene ‐Mediated SGN‐Like Neurons Differentiation and Functional Maturation

**FIGURE 5:**
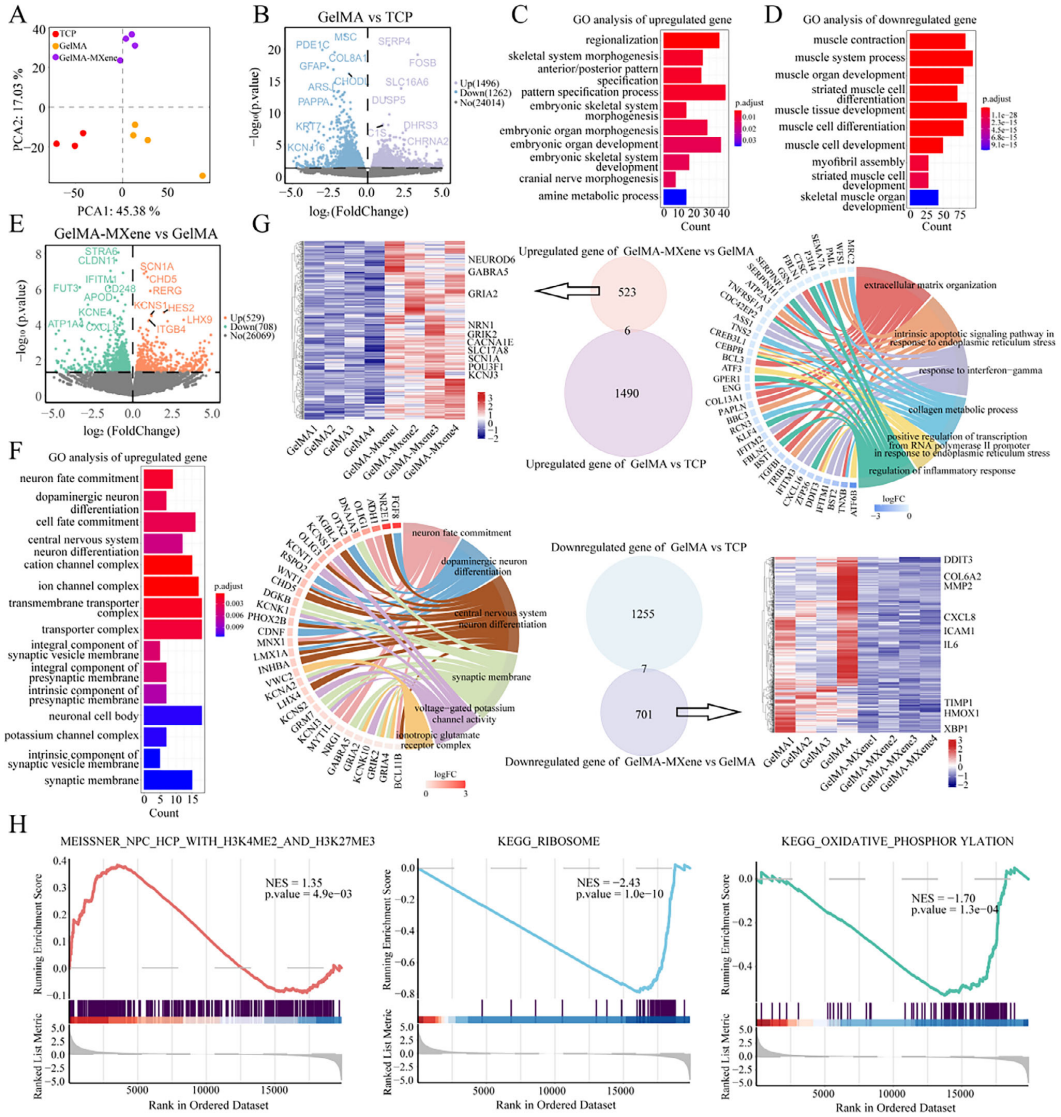
GelMA‐MXene scaffolds promote SGN‐like neurons maturation through transcriptomic reprogramming: (A) PCA showing distinct clustering of SGN‐like neurons cultured on TCP, GelMA, and GelMA‐MXene biomimetic scaffolds; (B) Volcano plot of DEGs between TCP and GelMA groups. (C,D) GO enrichment of upregulated; (C) and downregulated (D) DEGs in GelMA vs. TCP; (E) Volcano plot of DEGs between GelMA and GelMA‐MXene biomimetic scaffolds; (F) GO enrichment of upregulated DEGs in GelMA‐MXene vs. GelMA, highlighting neuronal differentiation and ion channel activity; (G) Left: Heatmap showing 523 uniquely upregulated differentially expressed genes (DEGs) in GelMA‐MXene biomimetic scaffolds compared with GelMA and TCP. Upper middle: Venn diagram illustrating the overlap and unique sets of upregulated DEGs. The 523 GelMA‐MXene‐specific genes were enriched in neuron fate commitment, dopaminergic differentiation, synaptic signaling, and ion channel activity (right, circular chord plot). Lower middle: Venn diagram of downregulated DEGs, showing 701 GelMA‐MXene‐specific genes associated with extracellular matrix remodeling, apoptotic signaling, and stress response. Right: Heatmap of representative GelMA‐MXene‐specific downregulated genes; (H) GSEA plots showing enrichment of neurogenic commitment signatures (left) and suppression of ribosome biogenesis and oxidative phosphorylation pathways (middle, right) in GelMA‐MXene biomimetic scaffolds compared with GelMA.

In order to explore the potential molecular mechanisms by which the GelMA‐MXene biomimetic scaffold influences the maturation of SGN‐like neurons, we performed an RNA sequencing analysis on SGN‐like neurons that were cultured on TCP, GelMA, and GelMA‐MXene biomimetic scaffolds. Global transcriptomic profiling revealed distinct clustering of SGN‐like neurons cultured on TCP, GelMA, and GelMA‐MXene scaffolds, as shown by PCA (Figure 5A). Compared with TCP, GelMA biomimetic scaffolds induced widespread but relatively nonspecific changes, with enrichment in developmental and morphogenetic processes alongside downregulation of cytoskeletal and contractile genes (Figure 5B–D). These alterations suggest that GelMA provides a supportive microenvironment for cellular remodeling, yet does not strongly bias SGN‐like neurons toward a neuronal maturation trajectory. By contrast, the addition of MXene resulted in a qualitatively distinct transcriptomic profile (Figure 5E). Notably, key neuronal fate and synaptic genes such as NEUROG2 [48], GRIA2 [49], and GABRA5 [50] were upregulated, while GO enrichment pointed to neuron fate commitment, dopaminergic differentiation, and ion channel activity (Figure 5F). Such shifts highlight the capacity of MXene to reinforce electrophysiological competence and lineage specification. Further analysis revealed 523 genes uniquely upregulated in GelMA‐MXene biomimetic scaffolds, enriched in synaptic signaling and neurotransmitter transport, whereas 701 uniquely downregulated genes were associated with stress responses, ECM remodeling, and apoptosis (Figure 5G). This reciprocal regulation indicates that MXene incorporation both enhances pro‐neurogenic pathways and attenuates inhibitory or stress‐related transcriptional programs. Consistently, Gene Set Enrichment Analysis (GSEA) demonstrated enrichment of neural progenitor commitment signatures and suppression of ribosome biogenesis and oxidative phosphorylation (Figure 5H), suggesting a metabolic shift from general biosynthesis to neuron‐specific specialization. Collectively, these findings demonstrate that MXene transforms GelMA from a passive scaffold into a bioactive matrix that orchestrates the maturation of SGN‐like neurons through coordinated transcriptional reprogramming.

### 2.6 Establishment of an In Vitro Humanized Auditory Circuit Model

**FIGURE 6:**
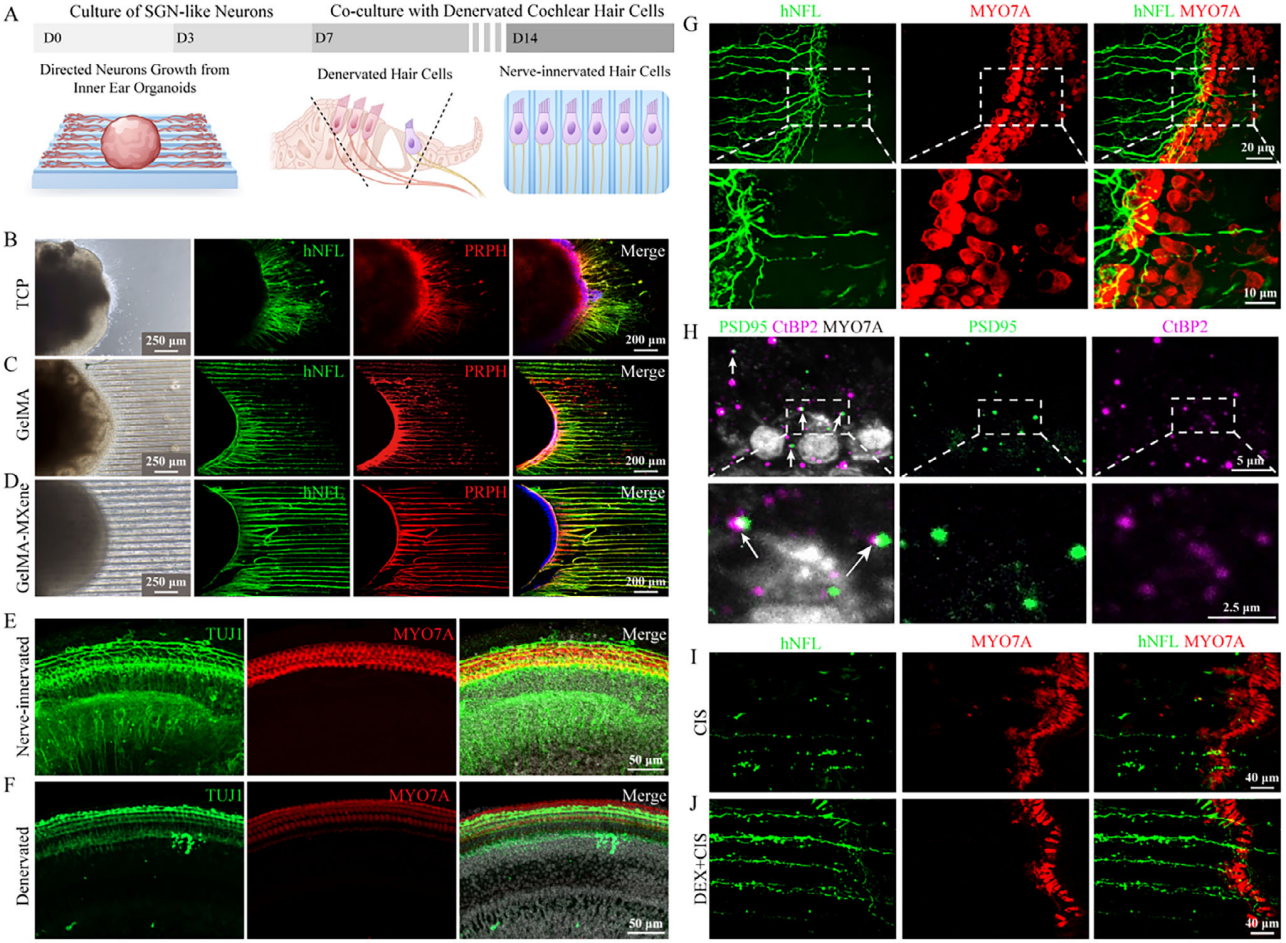
Human‐derived SGN‐like neurons integrate functional innervation in neural circuit: (A) Co‐cultivation experiment time flow chart; (B) Representative microscopic images illustrating the growth behavior of SGN‐like neurons derived from inner ear organoids on TCP. hNFL^+^ (green), PRPH^+^ (red); (C) Representative microscopic images illustrating the growth behavior of SGN‐like neurons derived from inner ear organoids on GelMA biomimetic scaffolds. TUJ1^+^ (green), PRPH^+^ (red); (D) Representative microscopic images illustrating the growth behavior of SGN‐like neurons derived from inner ear organoids on GelMA‐MXene biomimetic scaffolds. TUJ1^+^ (green), PRPH^+^ (red); (E) Representative microscopic images of normal mouse cochlear explants. MYO7A^+^ (red), TUJ1^+^ (green); (F) Representative microscopic images of denervated cochlear explants. MYO7A^+^ (red), TUJ1^+^ (green); (G) Immunofluorescence image of human inner ear organoids and co‐cultured cochlear basilar membranes after 7 days. Cochlear hair cells (MYO7A) are labeled in red, and hNFL are labeled in green; (H) Immunofluorescence image of synapse proteins after co‐culture of human inner ear organoids and cochlear basilar membranes for 14 days. CtBP2 is labeled in purple, PSD95 is labeled in green, and hair cells (MYO7A) are labeled in white; (I) Representative images of SGN‐like neurons and hair cells stained with immunofluorescence 24 h after cisplatin treatment in the co‐culture system. SGN‐like neurons are labeled with hNFL (green), and hair cells are labeled with MYO7A (red); (J) Representative immunofluorescence images from the co‐culture system show SGN‐like neurons that were subjected to 6 h DEX pretreatment prior to 24 h cisplatin treatment. SGN‐like neurons are labeled with hNFL (green), and hair cells are labeled with MYO7A (red).

To better simulate the hierarchical projections of the auditory circuit in vivo, we developed an in vitro co‐culture system based on a biomimetic scaffold that mimics the synaptic connections of the auditory system. By co‐culturing human‐derived SGN‐like neurons with the cochlear basilar membrane on the GelMA‐MXene scaffolds, we successfully established an auditory circuit in which cochlear hair cells project to auditory neurons (Figure 6A). Firstly, SGN‐like neurons migrating from inner ear organoids exhibit disorganized growth on TCP, however, they demonstrate directed outgrowth on the biomimetic scaffolds (Figure 6B–D). We then evaluated the potential cytotoxicity of the scaffolds toward cochlear hair cells and found that cell viability was preserved under these conditions (Figure S7). In addition, to eliminate the interference of endogenous SGNs during reinnervation, we used the mechanical approach to selectively remove SGNs from the cochlear basilar membrane. Consequently, all the neurons observed in the co‐culture system originated exclusively from human SGN‐like neurons. For the co‐culture experiments, we removed peripheral auditory nerve synapses from cochlear explants of FVB mouse, resulting in denervation while preserving hair cells largely intact (Figure 6E,F). Subsequently, we co‐cultured SGN‐like neurons with cochlear explants on biomimetic scaffolds. After 7 days of in vitro co‐culture, the axons extended toward the cochlear explants and established direct contact. Double immunolabeling with hair cell marker (MYO7A) and hNFL revealed that SGN‐like neurons axon terminals formed synaptic connections with MYO7A‐positive cells (Figure 6G). Consistent with synaptic patterns observed in adult inner ear tissue, synaptic contacts in the co‐culture system exhibited characteristic presynaptic‐postsynaptic pairing (CtBP2^+^, PSD95^+^) (Figure 6H). To evaluate the potential of our auditory circuit model for studying hearing loss, we created a drug‐induced injury model and assessed the efficacy of potential treatments. Prior studies have demonstrated that cisplatin impaired SGNs function and induced neuronal loss, ultimately leading to hearing loss [46, 51, 52]. Glucocorticoids (GCs) are widely used in clinical practice as immunomodulators and are employed in the treatment of various inner ear disorders, including ototoxic damage and drug‐induced hearing loss [53, 54]. Dexamethasone (DEX) is one of the most commonly used glucocorticoids and has demonstrated significant clinical efficacy in treating various types of hearing loss [55, 56]. To assess drug sensitivity and therapeutic responsiveness, SGN‐like neurons were treated with increasing concentrations of cisplatin (0, 7.5, 15, and 30 µM). Significant SGN‐like neurons loss was observed at 15 µM (Figure S8A). The SGN‐like neurons were then pretreated with dexamethasone (DEX) for 6 h before being exposed to cisplatin, with DEX maintained throughout the subsequent 24 h treatment period. Under these conditions, 80 nM DEX provided substantial protection for the SGN‐like neurons (Figure S8B). Furthermore, in the co‐culture system, the control group was treated with 15 µM cisplatin for 24 h (Figure 6I), while the experimental group was pretreated with DEX for 6 h prior to cisplatin treatment (Figure 6J). Immunofluorescence analysis revealed that pretreatment with DEX appeared to enhance neurite projection and contact with hair cells. In summary, our findings demonstrate that differentiated human SGN‐like neurons can form potential synaptic connectivity with cochlear hair cells. Therefore, compared with previous studies, a key advantage of our model is its ability to generate human‐derived SGN‐like neurons in vitro and promote directed axonal growth through conductive biomimetic scaffolds. This approach partly addresses the issue of random axonal outgrowth in SGN‐like neurons and more accurately simulates the physiological state of directed projections from SGNs to hair cells in vivo. However, the current model has several limitations, including the absence of vascular, immune, and other cochlear microenvironmental components. Consequently, it cannot fully recapitulate the complexity of the in vivo cochlear microenvironment.

## 3 Conclusions

In summary, we developed an effective method of culturing human‐derived SGN‐like neurons. Based on this, we used a topologically structured, electrically conductive biomaterial to create a human‐derived model of an auditory neural circuit in vitro. This engineered system supported survival, directional growth, and maturation of SGN‐like neurons, as well as promoting synaptic development and functional refinement. This is evidenced by increased expression of postsynaptic proteins and enhanced calcium signaling dynamics. Furthermore, transcriptomic analyses showed that the electrically conductive scaffolds modulate gene networks associated with neuronal maturation, synaptic organization and electrical activity. Notably, by integrating human SGN‐like neurons with cochlear hair cells in a controlled co‐culture environment, the reconstructed circuit faithfully modelled interactions between SGN‐like neurons and hair cells. Additionally, drug‐induced injury and protection assays demonstrated that this model reliably mimics hearing damage and therapeutic responses. Taken together, the auditory circuit model serves as a platform for studying the development of auditory circuits, synaptic connections, and mechanisms of degeneration. It can also serve as a platform for drug screening, particularly for assessing ototoxicity and evaluating potential auditory neuroprotective therapeutics.

## 4 Methods

### 4.1 Animals

All animal procedures were approved by the Animal Ethics Review Committee of Southeast University (Approval No. 20250108002) and were carried out in accordance with the relevant guidelines for the care of animals. FVB strain mice were used as wild‐type (WT) models in all experiments.

### 4.2 Synthesis of Ti_3_C_2_T_x_ MXene in Aqueous Solution

Initially, the reaction vessel was cleaned sequentially with NaOH and ddH_2_O, and then dried in an oven. The etching process commenced by mixing 1 g LiF with 10 mL HCl and 5 mL ddH_2_O until fully dissolved, followed by the slow addition of 1 g MAX powder. The mixture was then maintained in a 40°C water bath for 30 h with continuous agitation. To isolate the MXene, the crude product was centrifuged repeatedly, starting at 3500 rpm and increasing to 7500 rpm in 500 rpm intervals, with the supernatant discarded after each wash. During purification, the characteristic dark green product emerged and was collected by redispersing the sediment in ddH_2_O. The final concentration of the resulting MXene was determined by vacuum filtration overnight.

### 4.3 Reparation of GelMA‐MXene Electrically Conductive Biomimetic Scaffolds

Gelatin (20 g) was dissolved in 200 mL of preheated PBS (60°C) under continuous stirring. Methacrylic anhydride (MA) was then added dropwise to initiate the methacrylation reaction. To eliminate the unreacted MA, the crude solution was transferred to 50 mL tubes and centrifuged. The desired supernatant was then recovered for further processing. Subsequently, 400 mL of deionized water preheated to 40°C was added to the supernatant. This solution was then transferred to a dialysis bag and dialyzed against deionized water at 45°C with continuous stirring for seven days. After dialysis, the pH was adjusted to neutral and the solution freeze‐dried to obtain solid GelMA. The prepolymer was prepared by dissolving GelMA in PBS, supplemented with 1% (v/v) HMPP as a photoinitiator, to achieve a final concentration of 15 wt.%. Subsequently, various dosages of MXene (100–500 µg/mL) were incorporated into the GelMA matrix. The resulting precursor mixtures were then patterned through a photomask to generate biomimetic scaffolds with grooved topographies.

### 4.4 Characterizations

Samples were first pre‐frozen at −20°C and subsequently lyophilized for 24 h using a freeze dryer. The dried specimens were then coated with a conductive metal layer via sputter coating (six cycles of 10 s each). The microgroove architecture was observed by SEM (Hitachi S‐3000N), while the morphology of MXene nanosheets was further analyzed using TEM. Raman spectra were collected with a Raman microscope. Surface elemental composition and chemical states were determined by XPS, and crystalline structures were characterized using XRD. Mechanical properties were measured with a universal testing machine.

### 4.5 Cytotoxicity Assays

The cytotoxicity of the biomimetic scaffolds was determined using a CCK‐8 assay after culturing SGN‐like neurons on GelMA‐MXene substrates (0–500 µg/mL MXene) for 72 h. For the assay, the original culture media were replaced with fresh media containing 1% (v/v) CCK‐8 reagent. After 2 h incubation at 37°C, the optical density (OD) was recorded at a wavelength of 450 nm using a microplate reader.

### 4.6 Cell Culture

The hiPSCs were dissociated into single cells, which were then seeded into 96‐well U‐bottom plates to form aggregates. Inner ear organoid differentiation was induced by modulating key developmental signaling pathways sequentially. In brief, the cells were cultured in an E6 medium (Thermo Fisher, A1516401) containing BMP4 (ReproCELL, 03–0007), FGF‐2 (Stem Cell Technologies, 780003), SB431542 (Stemgent, 04‐0010‐05), and Matrigel (Corning, 354230) during the initial stage of induction. This was followed by treatment with LDN‐193189 (Stemgent, 04‐0074‐02) and CHIR99021 (ReproCELL, 04‐0004‐10) to promote otic lineage specification. Subsequently, organoid maturation was supported using Organoid Maturation Medium (OMM) supplemented with CHIR99021 and Purmorphamine (Stemgent, 04–0009), and later IWP‐2 (Tocris Bioscience, 3533), to further promote inner ear sensory and neuronal differentiation.

After culturing the inner ear organoids for 30 days, they were digested with TrypLE (Thermo Fisher, 12604013) in a 37°C, 5% CO_2_ incubator for 15 min, and gently pipetted to fully dissociate the organoids into a single‐cell suspension. The cells were then centrifuged at 1000 rpm for 3 min, the supernatant was discarded, and the cells were resuspended in OMM medium. Finally, the dissociated cells were seeded onto Matrigel‐precoated slides and biomimetic scaffolds, which were then placed in a 24‐well plate and cultured further in a 37°C, 5% CO_2_ incubator for subsequent experimental analysis. OMM was prepared by mixing Advanced DMEM/F12 (Thermo Fisher, 12634028) and Neurobasal medium (Thermo Fisher, 21103049) in a 1:1 ratio, supplemented with 0.5x N2 supplement (Thermo Fisher, 17502048), 1x B27 supplement (without vitamin A; Thermo Fisher, 12587010), 1x GlutaMAX (Thermo Fisher, 35050061), 0.1 mM β‐mercaptoethanol (Thermo Fisher, 21985023), and Normocin (Invivogen, ant‐nr‐05).

### 4.7 Real‐time Quantitative Polymerase Chain Reaction (RT‐qPCR)

SGN‐like neurons were maintained on TCP, GelMA, or GelMA‐MXene scaffolds for up to 14 days (D14), after which cells were collected, lysed, and processed for RNA isolation using a commercial kit (Vazyme, RC401‐01). The purified mRNA was subsequently reverse‐transcribed into cDNA with cDNA synthesis kit (Vazyme, R233‐01). The cDNA was either used immediately for qPCR analysis or stored at −80°C until use. Relative gene expression levels were determined using the 2^−ΔΔCt^ method. The qPCR primer sequences were as follows Table 1:

**TABLE 1:** The qPCR primer sequences.

Gene	Forward primer (5’‐3’)	Reverse primer (5’‐3’)
PROX1	AGCAAATGACTTTGAGGTTCCA	CTCTTGTAGGCAGTTCGGGG
TUJ1	ACCAGATCGGGGCCAAGTT	GAGGCACGTACTTGTGAGAAGA
PRPH	TAAAGACGACTGTGCCTGAG	GGATGCCTGGTACCACCTTT
BRN3A	CACCATCTGCAGGTTCGAGT	TTGAGGTCCAGTTTCTCGGC
GLUA2	AGAGTGCGGAAGTCCAAAGG	GGCACTGGTCTTTTCCTTACT
VGUT3	TCACATCTTTGCCGGTTTATGC	GCTGACAAGAGACCCACCTTA
ISL	CAACAAACAAAACGCAAAAC	AAGTCAAACACAATCCCGA
NEUROD1	ATGACCAAATCGTACAGCGAG	GTTCATGGCTTCGAGGTCGT
GAPDH	TCGGAGTCAACGGATTTGGT	TTCCCGTTCTCAGCCTTGAC

### 4.8 Immunofluorescence

Following fixation in 4% PFA and permeabilization with PBST, cells were blocked in 0.5% FBS at room temperature. Primary antibody incubation was carried out overnight at 4°C, after which samples were equilibrated for 30 min and washed with PBS. Secondary antibodies and DAPI were then applied for 1 h. After three final PBS washes, the samples were mounted and imaged using Zeiss confocal microscopy.

### 4.9 Calcium Imaging

After 14 days of in vitro culture, SGN‐like neurons were incubated with Fluo‐4 AM (Beyotime, S1060) at a 1:1000 dilution in culture medium. After 30 min of at 37°C, the cells were rinsed thrice with PBS. The resulting samples were then retained in a PBS environment for subsequent fluorescence imaging. Imaging was performed using an inverted microscope (Olympus) equipped with 10× objective. Time‐lapse images were acquired at 900 ms intervals for 3 min within a single field of view, at a resolution of 512 × 512 pixels. Changes in fluorescence intensity within regions of interest (ROIs) and the background were quantified over time using ImageJ software. Relative fluorescence intensity (ΔF) was calculated as the ratio of the fluorescence intensity within the ROI to that of the background.

### 4.10 Transcriptome Sequencing

Total RNA extraction was executed utilizing TRIzol lysis buffer (Thermo Fisher Scientific, 15596026CN) in accordance with the manufacturer's protocol. Subsequently, cDNA libraries were constructed and sequenced at Novogene in China using the Illumina NovaSeq X Plus system. The DESeq2 R package was employed for bioinformatic analysis to identify differentially expressed genes (DEGs) from the count matrix, with a significance threshold set at *p *< 0.05. Transcript levels were quantified and normalized as fragments per kilobase of transcript per million mapped fragments (FPKM). Finally, the R‐based tool pheatmap was used to visualize the FPKM data via heatmaps.

### 4.11 Preparation of Denervated Organs of Corti for Culture

Denervated cochlear explants were prepared using the method described above [12]. In brief, using micromanipulation, a blade was employed to dissect near the base of the inner hair cells, separating the cochlear tissue, which contains intact inner and outer hair cell rows and surrounding supporting cells, from the attached SGNs, thereby obtaining denervated cochlear explants. Subsequently, the explants were seeded onto Matrigel‐coated glass slides and placed in a 37°C, 5% CO_2_ incubator for further culture.

### 4.12 Co‐Culture of SGN‐Like Neurons and Cochlear Explants

Under sterile conditions, the basilar membranes of postnatal day 2–3 (P2‐P3) FVB mice were carefully dissected and transferred onto Matrigel‐coated glass slides and biomimetic scaffolds. After allowing the cochlear explants to adhere slightly and air‐dry briefly, the culture medium was added. This consisted of DMEM/F12 (Gibco, 11330‐032), supplemented with 2% B27 (Gibco, 17504044), 1% N‐2 supplement (Gibco, A1370701), and 50 µg/mL ampicillin (Beyotime, ST008). The cochlear explants were then maintained at 37°C in a humidified incubator containing 5% CO_2_. Following an overnight pre‐culture period, inner ear organoids were seeded onto the glass slides and allowed to fully attach. This was followed by continued co‐culture for 5–14 days, after which the organoids were subjected to subsequent experimental analyses.

### 4.13 Data Statistics and Analysis

ImageJ software was used to analyze the lengths and growth directions of SGN‐like neurons’ neurites, with the horizontal direction to the right defined as 0° and angles measured in a clockwise direction. Image‐Pro Plus software was used to quantify the areas of neurite growth cones, average filopodia lengths, filopodia counts, and synaptic densities. All experiments were conducted with at least three independent biological replicates. Cell morphological parameters were quantified using ImageJ. Quantitative data were analyzed and plotted using GraphPad Prism 6. Quantitative data are reported as the mean ± SD. Statistical significance was verified using a one‐way analysis of variance (ANOVA) or an unpaired two‐tailed Student's *t*‐test. Statistical significance was defined as ^*^ *p* ≤ 0.05, ^**^ *p* ≤ 0.01, ^***^ *p* ≤ 0.001, with *p* > 0.05 considered not significant (ns.). Final figure layouts were polished using Adobe Illustrator.

## Author Contributions

Y.N.H., R.J.C., M.H.L., L.T. conceived the idea and designed the experiment. P.F., Q.Z., Y.S.W., H.R. designed and conducted experiments, analyzed the data, and wrote the manuscript. X.Z., F.C.W., T.Q.Y., assisted with data analysis. W.X.W., J.L. assisted with cell culture.

## Conflicts of Interest

The authors declare no conflicts of interest.

## Supporting information




**Supporting File**: advs76525‐sup‐0001‐SuppMat.docx.

## Data Availability

The data generated and analyzed during the current study are available from the corresponding author upon reasonable request.
