# Supplementary material for: Functionalized Biomimetic Scaffolds for Human‐Derived Auditory Neural Circuit Construction
Source: Adv Sci (Weinh). 2026 Jul 11:e76525. Online ahead of print. doi: 10.1002/advs.76525 (PMC13355911; doi:10.1002/advs.76525)
Supplement: Supplementary file 1 — Supporting File: advs76525‐sup‐0001‐SuppMat.docx. [file ADVS-9999-e76525-s001.docx]

**Supplementary**

**Functionalized Biomimetic Scaffolds for Human-Derived Auditory Neural Circuit Construction**

Pan Feng^1,#^, Qian Zhu^1,2,#^, Yusong Wang^1,#^, Hao Rong^3,#^, Xu Zhang^1^, Fuchun Wang^1^, Tianqi Yu^1^, Wenxuan Wang^1^, Jing Li^1^, Lei Tian^1,*^, Menghui Liao^4,*^, Renjie Chai^1,5,6,*^, Yangnan Hu^1,4,*^

1. Spine Surgery Department, Nantong First People’s Hospital, State Key Laboratory of Digital Medical Engineering, Jiangsu Provincial Key Laboratory of Critical Care Medicine, School of Life Sciences and Technology, School of Medicine, Advanced Institute for Life and Health, Southeast University, Nanjing 210096, China.
2. Department of Rehabilitation Medicine, Zhongda Hospital, Southeast University, Nanjing, 210009, China.
3. The affiliated Lihuili Hospital of Ningbo University, Ningbo, China, 315000.
4. School of Medical Engineering, Affiliated Zhuhai People’s Hospital, Beijing Institute of Technology, Zhuhai, 519088, China.
5. Co-Innovation Center of Neuroregeneration, Nantong University, Nantong 226001, China.
6. Department of Neurology, Aerospace Center Hospital, School of Life Science, Beijing Institute of Technology, Beijing 100081, China.

#: These authors contributed equally to this work

***Corresponding Authors:**

Email: yangnanhu@bit.edu.cn (Yangnan Hu); renjiec@seu.edu.cn (Renjie Chai); menghuiliao@bit.edu.cn (Menghui Liao); tianlei@seu.edu.cn (Lei Tian).


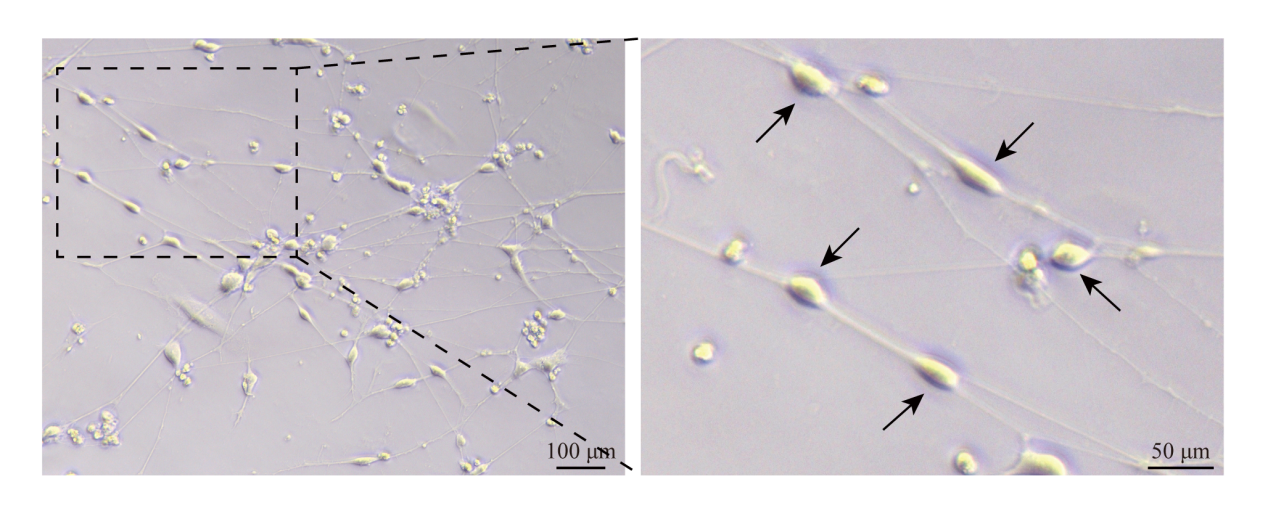


**FIGURE S1.** Bright field representative image of hiPSC-derived SGN-like neurons after three days of TCP culture. SGN-like neurons show a distinct bipolar neuron morphology.


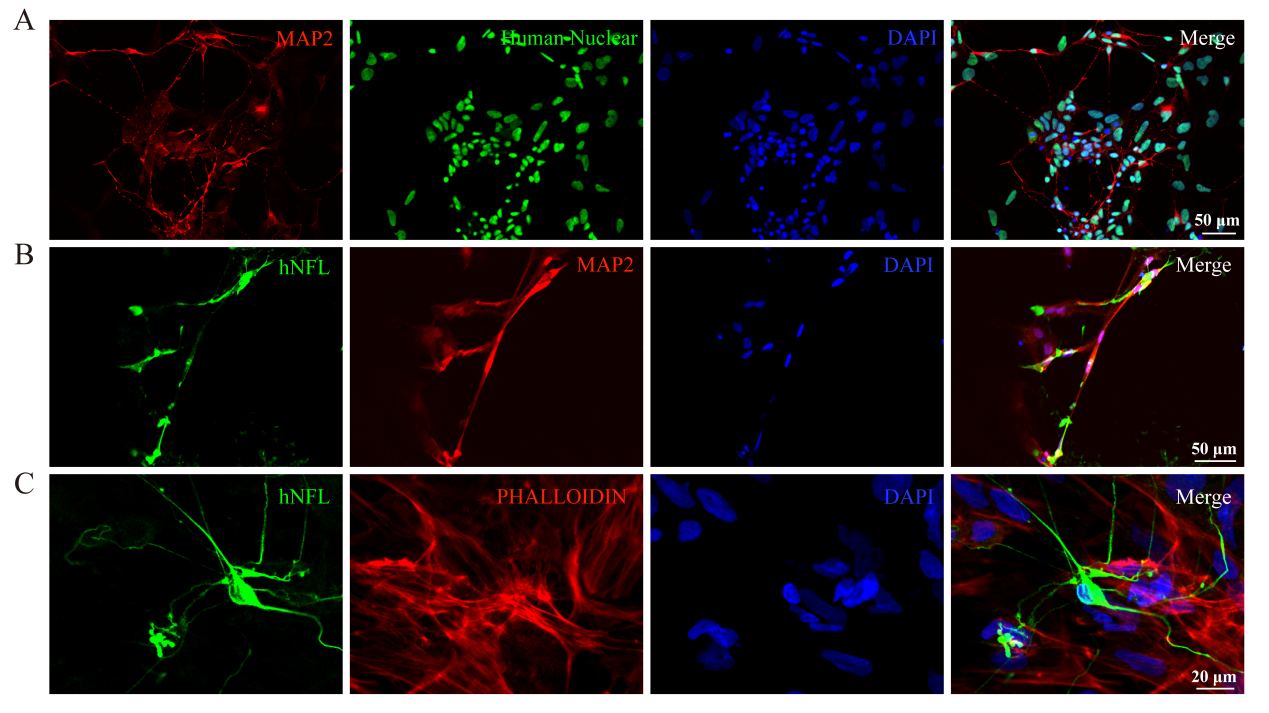


**FIGURE S2.** (A) Representative immunofluorescence images of mature neuronal marker MAP2^+^ (red), Human Nuclear^+^ (green), and cell nucleus DAPI^+^ (blue) 3 days after hiPSC-derived SGN-like neurons were seeded on TCP; (B) Representative immunofluorescence images of human neurofilament protein (hNFL, green), the broad-spectrum mature neuron marker MAP2 (red), and cell nuclei (DAPI, blue) 3 days after inoculation of hiPSC-derived SGN-like neurons onto TCP; (C) Representative confocal images showing hNFL and Phalloidin staining of SGN-like neurons cultured on TCP, hNFL^+^ (green), PHALLOIDIN^+^ (red), DAPI^+^ (blue).


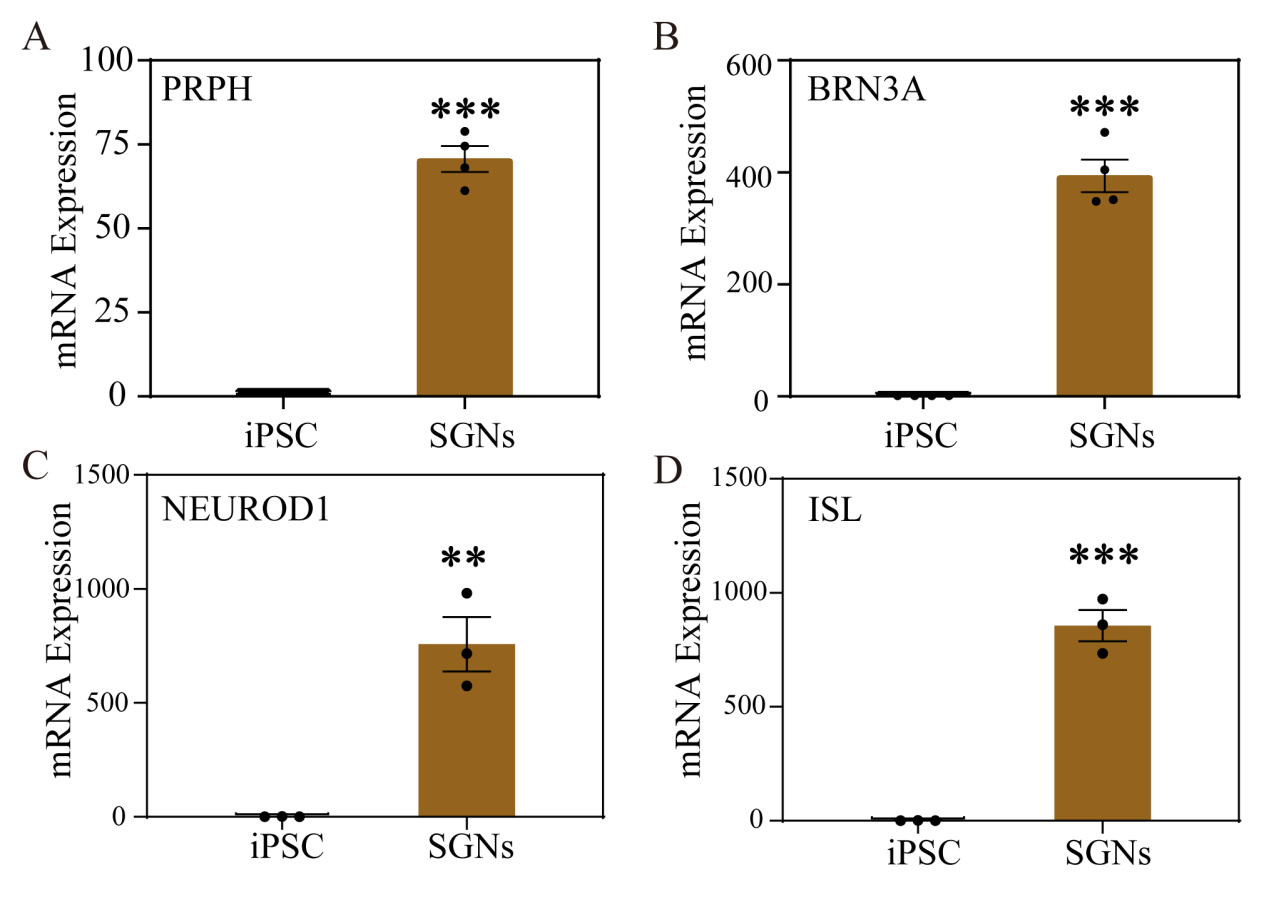


**FIGURE S3.** (A) qPCR validation of PRPH gene expression levels in SGN-like neurons and hiPSCs; (B) qPCR validation of BRN3A gene expression levels in SGN-like neurons and hiPSCs; (C) qPCR validation of NEUROD1 gene expression levels in SGN-like neurons and hiPSCs; (D) qPCR validation of ISL gene expression levels in SGN-like neurons and hiPSCs. Data points illustrate the mean ± standard deviation (SD). *P*-values are categorized as follows: ***P* < 0.01, ****P* < 0.001 and non-significant differences (*P* > 0.05) are designated as ns.


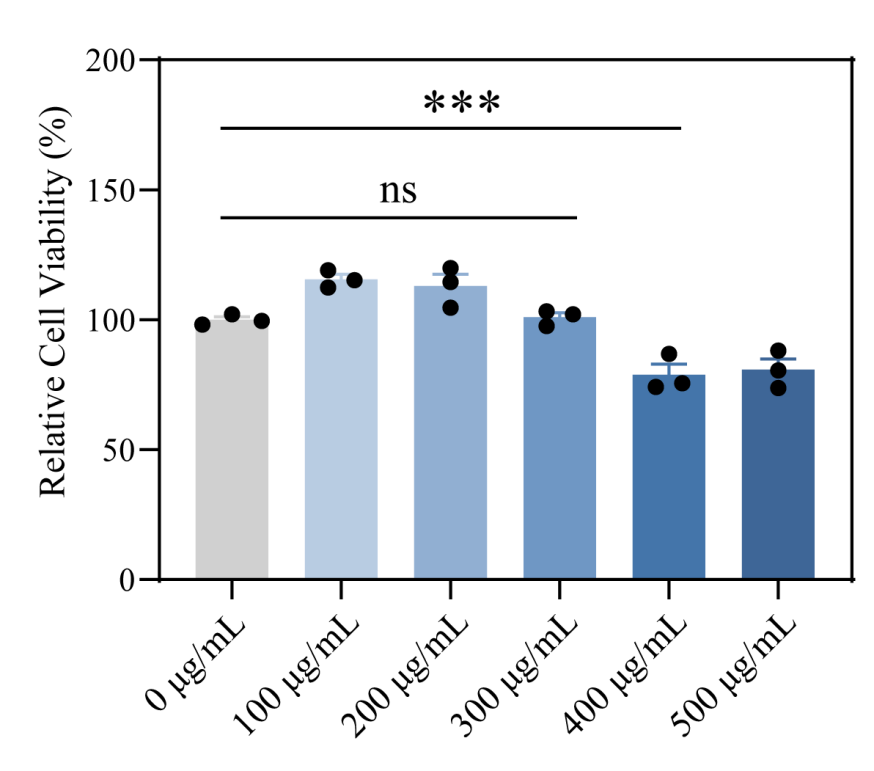


**FIGURE S4.** The viability of SGN-like neurons was assessed via CCK-8 analysis after the cells had been cultured on GelMA-MXene biomimetic scaffolds containing varying concentrations of MXene (0-500 µg/mL) for three days. Data points illustrate the mean ± standard deviation (SD). *P*-values are categorized as follows: ****P* < 0.001 and non-significant differences (*P* > 0.05) are designated as ns.


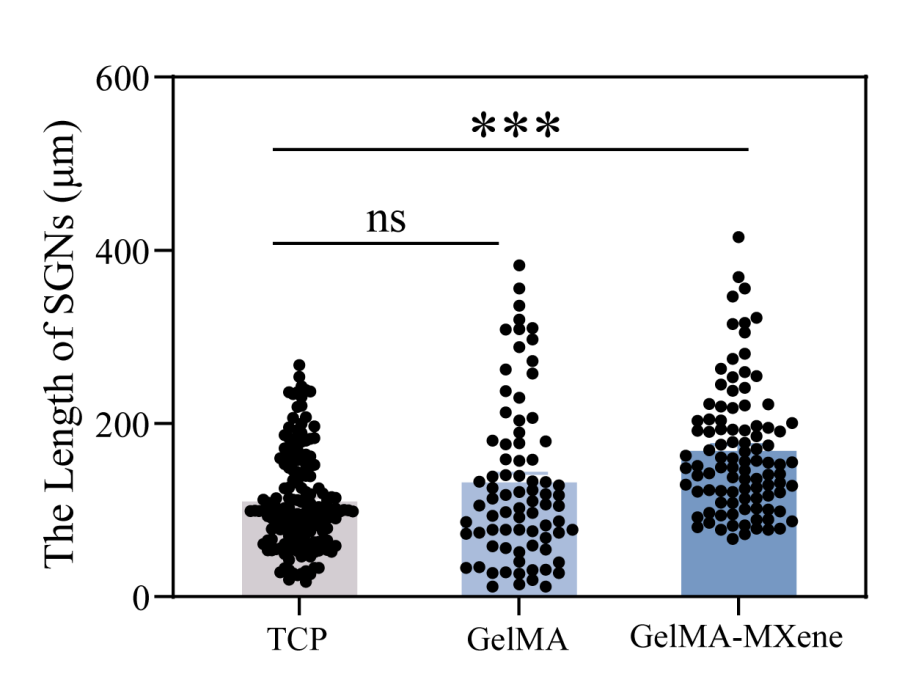


**FIGURE S5.** GelMA-MXene biomimetic scaffolds promote the elongation of SGN-like neurons. We cultured SGN-like neurons on TCP, GelMA, and GelMA-MXene biomimetic scaffolds. After 3 days of *in vitro* culture, the axonal extension length of SGN-like neurons on the GelMA-MXene scaffolds significantly increased compared to the TCP and GelMA groups. Data points illustrate the mean ± standard deviation (SD). *P*-values are categorized as follows: ****P* < 0.001 and non-significant differences (*P* > 0.05) are designated as ns


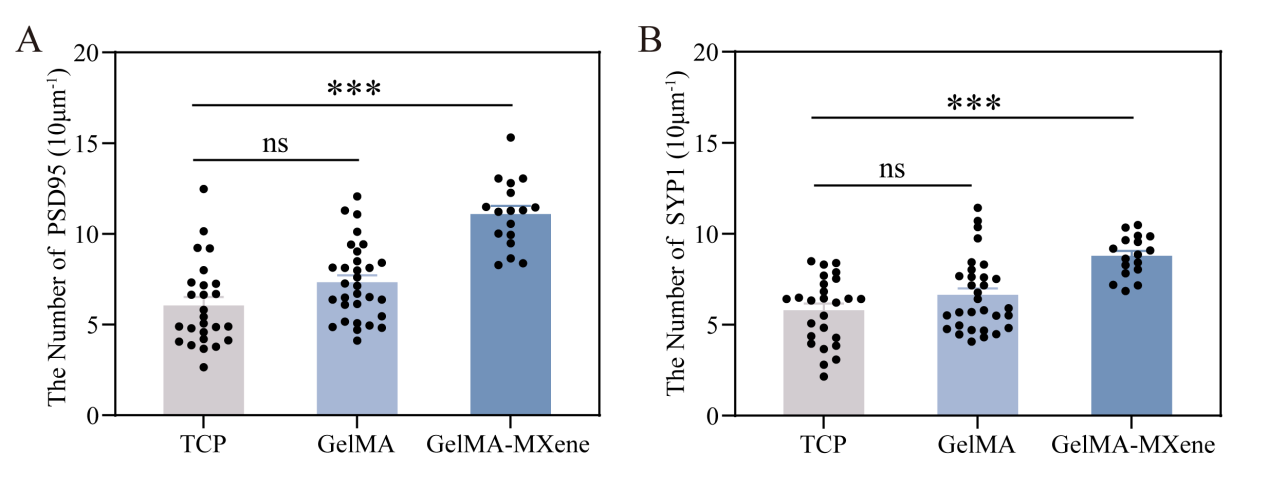


**FIGURE S6.** GelMA-MXene biomimetic scaffolds promote the synaptic development of SGN-like neurons: (A) Quantitative assessment of the number of PSD95^+^ puncta per 10 μm of dendritic segment; (B) Quantitative assessment of the number of SYP1^+^ per 10 μm of dendritic segment. Data points illustrate the mean ± standard deviation (SD). *P*-values are categorized as follows: ****P* < 0.001 and non-significant differences (*P* > 0.05) are designated as ns.


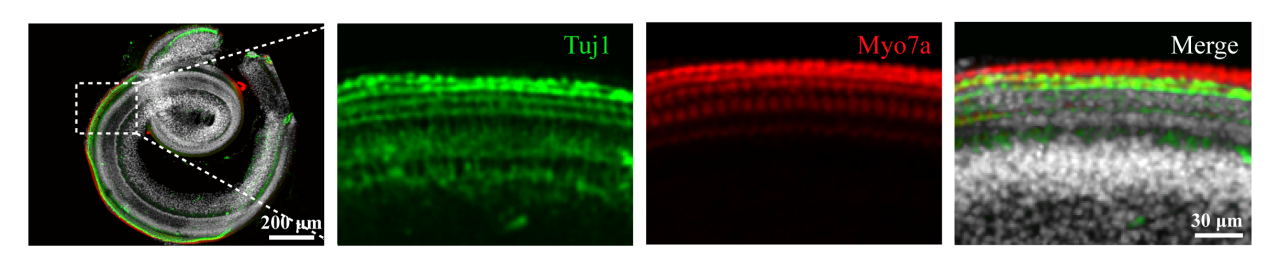


**FIGURE S7.** Representative microscopic images of immunofluorescence staining after 7 days of culture of mouse cochlear explants on biomimetic scaffolds. MYO7A (red) marks hair cells, TUJ1(green) marks SGNs, and DAPI (white).


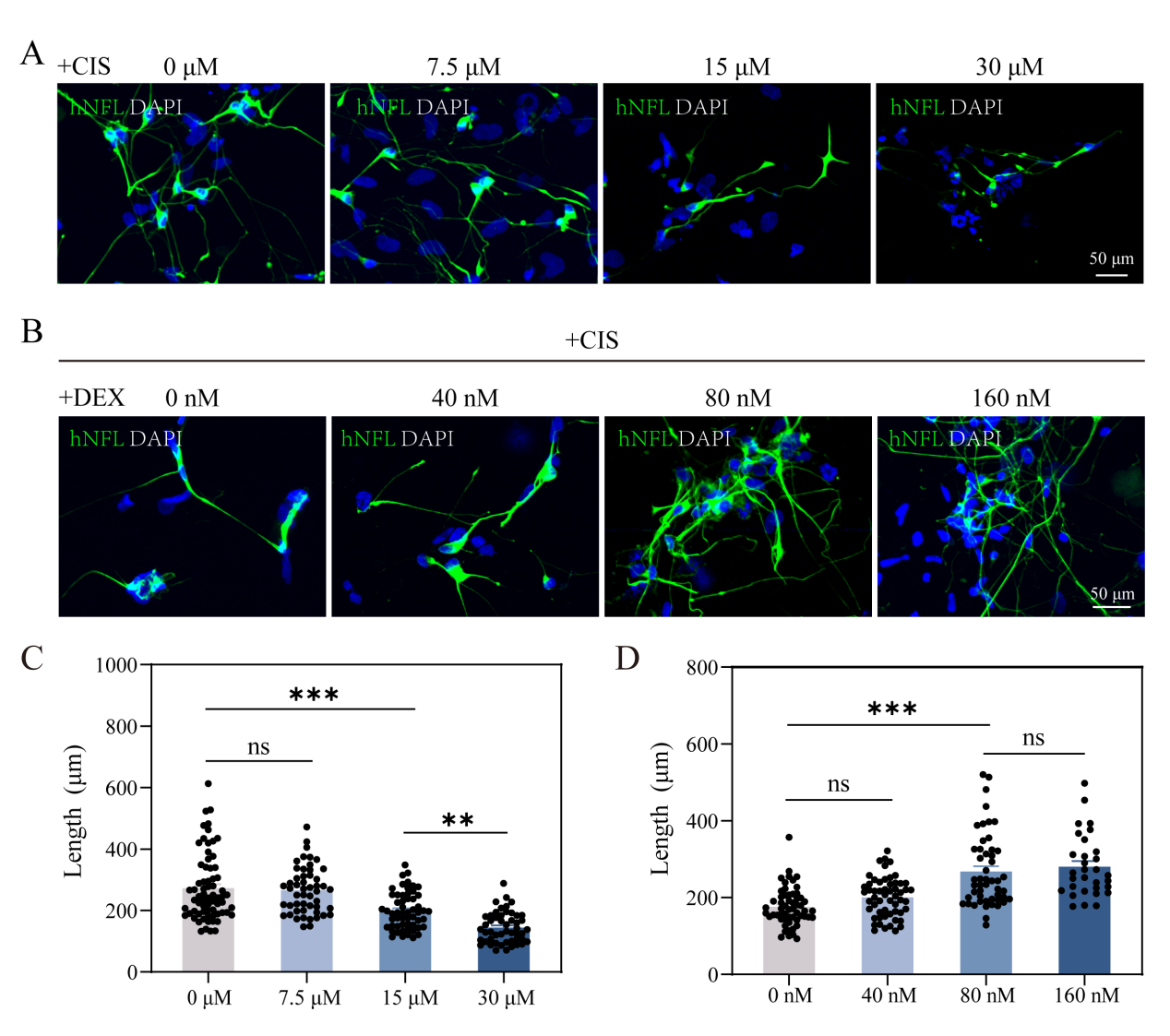


**FIGURE S8.** Efficacy assessment and functional validation of humanized neural circuit models: (A) SGN-like neurons were treated with cisplatin (0-30 µM) for 24 h and analyzed by immunofluorescence hNFL, green; DAPI, blue).; (B) The protective effects of dexamethasone (DEX) against cisplatin toxicity were investigated. SGN-like neurons were first pretreated with DEX (0-160 nM) for 6 hours, followed by 24-hour stimulation with CIS and DEX. Immunostaining was then performed to analyze neuronal morphology using hNFL (green) and DAPI (blue). (C) Statistical quantification of SGN-like neurite length following cisplatin-induced injury. (D) Quantitative analysis of SGN-like neurite length following dexamethasone pretreatment and cisplatin-induced injury. Data points illustrate the mean ± standard deviation (SD). *P*-values are categorized as follows: ***P* < 0.01, ****P* < 0.001 and non-significant differences (*P* > 0.05) are designated as ns.
